# What Does the Future Hold for Yellow Fever Virus? (II)

**DOI:** 10.3390/genes9090425

**Published:** 2018-08-21

**Authors:** Raphaëlle Klitting, Carlo Fischer, Jan F. Drexler, Ernest A. Gould, David Roiz, Christophe Paupy, Xavier de Lamballerie

**Affiliations:** 1 Unité des Virus Émergents (UVE: Aix-Marseille Univ–IRD 190–Inserm 1207–IHU Méditerranée Infection), 13385 Marseille CEDEX 05, France; eag@ceh.ac.uk (E.A.G.); xavier.de-lamballerie@univ-amu.fr (X.d.L.); 2 Charité-Universitätsmedizin Berlin, Freie Universität Berlin, Humboldt-Universität zu Berlin, and Berlin Institute of Health, Institute of Virology, 10117 Berlin, Germany; carlo.fischer@charite.de (C.F.); felix.drexler@charite.de (J.F.D.); 3 German Center for Infection Research (DZIF), 38124 Braunschweig, Germany; 4 Martsinovsky Institute of Medical Parasitology, Tropical and Vector Borne Diseases, Sechenov University, 119991 Moscow, Russia; 5 UMR Maladies Infectieuses et Vecteurs: Écologie, Génétique Évolution et Contrôle (MIVEGEC: IRD, CNRS, Univ. Montpellier), 34394 Montpellier, France; david.roiz@ird.fr (D.R.); christophe.paupy@ird.fr (C.P.)

**Keywords:** yellow fever virus, flavivirus, vector-borne transmission, emergence

## Abstract

As revealed by the recent resurgence of yellow fever virus (YFV) activity in the tropical regions of Africa and South America, YFV control measures need urgent rethinking. Over the last decade, most reported outbreaks occurred in, or eventually reached, areas with low vaccination coverage but that are suitable for virus transmission, with an unprecedented risk of expansion to densely populated territories in Africa, South America and Asia. As reflected in the World Health Organization’s initiative launched in 2017, it is high time to strengthen epidemiological surveillance to monitor accurately viral dissemination, and redefine vaccination recommendation areas. Vector-control and immunisation measures need to be adapted and vaccine manufacturing must be reconciled with an increasing demand. We will have to face more yellow fever (YF) cases in the upcoming years. Hence, improving disease management through the development of efficient treatments will prove most beneficial. Undoubtedly, these developments will require in-depth descriptions of YFV biology at molecular, physiological and ecological levels. This second section of a two-part review describes the current state of knowledge and gaps regarding the molecular biology of YFV, along with an overview of the tools that can be used to manage the disease at the individual, local and global levels.

## 1. Introduction, a Re-Emerging Arboviral Haemorrhagic Fever

Arthropod-borne viruses (“arboviruses”) are pathogens that are transmitted to their vertebrate hosts by arthropod vectors. This group includes four families, namely, *Togaviridae*, *Bunyaviridae*, *Flaviviridae* and *Reoviridae*. Viral haemorrhagic fevers (VHFs) are serious diseases caused by infection with viruses from several families: *Arenaviridae*, *Filoviridae*, *Flaviviridae* and members of the former *Bunyaviridae* family (e.g., *Nairoviridae*, *Peribunyaviridae*). Infections with haemorrhagic fever viruses are characterised by the development of fever with severe symptoms that can progress to shock and death in infected patients. Yellow fever virus (YFV) belongs to the *Flavivirus* genus (*Flaviviridae* family) and lies in the overlap area of arboviral diseases and VHFs. In Africa, YFV is primarily transmitted to humans by domestic urban-dwelling *Aedes aegypti* mosquitoes, whereas in South America, most human infections arise from spillover ensured by sylvatic *Aedes*, *Haemagogus* and *Sabethes* mosquitoes that previously fed on infected non-human primates (NHPs). In humans, YFV infection can cause severe disease, often accompanied by haemorrhagic symptoms, with a high case-fatality rate ranging between 20 and 50% [1].

In humans, wild-type YFV infection is primarily viscerotropic and affects the liver before damaging other tissues, including kidneys, spleen, lymph nodes and heart. The clinical presentation of the infection ranges from inapparent cases to severe—and sometimes fatal—haemorrhagic diseases, with a ratio of inapparent to apparent infection around 7–12:1 (estimated from fields studies in Africa) [2]. In the case of apparent Yellow fever (YF) infections, three phases have been described: After an incubation period of three to six days, the “infection” phase is characterised by a flu-like illness starting with an abrupt onset of fever accompanied by chills, headache, nausea and myalgia [3,4,5]. The peak of viraemia occurs during this first phase, around three days after the onset of the symptoms [2]. Then follows a “remission” period after which most infected individuals recover. However, in severe forms, after this brief period (up to 48 h) of symptoms subsidence, patients progress to the intoxication phase. At this third stage, the haemorrhagic and hepatic signs of illness occur, along with multi-organ dysfunction. This intoxication phase is accompanied by symptoms characteristic of YF disease that include jaundice (which gave YF its name), vomiting (black vomit or vomito negro, other former names of the disease) and other haemorrhagic manifestations such as vascular leakage. This is also the stage at which the first circulating, YF-specific antibodies can be detected in the blood [6]. For individuals that survive the “intoxication” phase, the convalescence that follows occasionally involves hepatitis and other associated symptoms and may last for months [7].

Much of our knowledge regarding YF disease arose from the combination of data from occasional clinical assessments and studies in experimental models, of which only a limited number were achieved in macaques [6,8]. Hence, our understanding of YFV pathogenesis remains scarce and there is still a lot to be understood regarding the factors that determine the course of YFV infection.

After the infecting bite, the virus encounters and infects dendritic cells in subcutaneous tissues. They ensure its delivery to the lymph nodes, where it replicates and initiates the cellular immune response. After this first round of replication, the virus is released into the blood circulation through the lymphatic ducts (viraemia) and spreads to several organs notably the liver, heart and kidneys [8,9,10]. The liver is the primary site of amplification for YFV and is thus central in the establishment of the disease. There, viral replication causes apoptosis and necrosis in hepatocytes, resulting in severe liver damage, a major component of viral pathogenesis [11]. YFV appears to have a broad tissue tropism and can disseminate to other organs [12,13,14,15,16,17]. It is likely that viral replication plays a role in the development of the extra-hepatic injuries that can be observed in the heart, thymus, kidney, and spleen. However, this cause-and-effect relationship remains to be formally demonstrated.

Also, the immune response to YFV infection is thought to contribute to pathogenesis under the form of a “cytokine storm”. Elevated patterns of pro- and anti-inflammatory cytokines were observed in patients with fatal YF, whereas patients with non-fatal YF—and notably without haemorrhagic manifestations—only had anti-inflammatory cytokine elevations [18,19,20]. Such an exuberant immune response resembles that observed in case of bacterial sepsis or severe filoviral infections and may contribute to the toxicity observed in the third phase of YF disease, notably the vascular leakage and hepatotoxicity [2,18,19,20,21,22].

As one might expect for a live-attenuated vaccine of which 20 to 60 million doses are distributed each year, the biology and notably the determinants of YFV 17D strain attenuation have been carefully examined since the elaboration of the vaccinal strain (e.g., [11,12,13,14,23,24,25,26,27]). For this reason, and also likely because the vaccine strain represents a convenient model in terms of biosafety, a good part of our understanding of YFV biology arose from studies based on the YFV 17D strain, and we still have much to learn about wild-type YF yellow fever viruses. Notably, only limited experimental data are available regarding the viral factors that determine the clinical outcome of YFV infections and our knowledge is scarce regarding the association between the genetic and phenotypic profiles of wild-type YFV strains [28,29]. Several studies have reported differences in replicative fitness and virulence among YFV strains from different genotypes [30,31,32,33,34], but the causal link between specific mutations/lineages and definite phenotypic features (e.g., virulence, viral fitness, vector-adaptation) was rarely investigated [31,35]. Finally, an important gap remains to be filled regarding the study of intrahost sequence variability in YFV isolates and its significance to viral replicative fitness, virulence or vector-competence [28,36].

From the 17th to the 19th centuries, YFV was considered a plague, as it caused deadly and often wide-ranging epidemics in Africa, the Caribbean, South and North America and even Europe. Thanks to the considerable investigation efforts that were made from the end of the 19th century, the mode of transmission of the virus was discovered as early as 1900 [37] and by the year 1937, the extremely efficacious, live-attenuated, YFV 17D vaccine was already in use [2,38]. Following these discoveries, in the 1940s–1950s, large vector-control and immunisation campaigns were implemented and proved to be extremely efficient in containing YFV outbreaks in endemic areas of Africa and Latin America [39]. Notably, thanks to carefully controlled eradication plans against the urban vector, *A*. *aegypti*, urban YFV was wiped out in numerous countries of the Caribbean, Central and South America such as Cuba [40]. De facto, by the end of the 20th century, YFV had turn from a plague to a disease of minor importance in Public Health.

This is probably one of the reasons why our understanding of the molecular biology, ecology and pathogenesis of YFV remains relatively limited given its historical and medical importance and as compared to other members of the *Flavivirus* genus. However, since the 2000s, there has been an upsurge of YF across Africa and most recently in Angola and the Democratic Republic of the Congo (DRC), with ~962 cases recorded between 2015 and 2016, and several exported cases, including to China [41]. Almost immediately afterwards, one of the largest YFV outbreaks in decades shook Brazil, causing ~2010 human cases and, alarmingly, revealing the extension of YFV’s area of endemicity near to the densely populated urban centres, Rio de Janeiro and Sao Paulo [42]. Currently, one African outbreak is still active in Nigeria, where 1903 cases have been reported, of which 108 have been confirmed (presumptive positives) [43]. These resurgence events clearly indicate that we do not have full control over YFV activity and they bring us back to the intricate and pivotal questions of the likeliness of occurrence of large urban YFV outbreaks in the Americas and of a successful import of YFV to Asia. Efforts have to be made to improve our understanding of YFV biology and to establish adequate measures to control the spread of this disease in and outside endemic areas. While the first portion of this two-part review outlined the main aspects of YFV ecology, phylogeny, and recent epidemiology, this second part will be dedicated to the current state of knowledge of the molecular biology of the virus, along with an overview of the tools that can be used to manage the disease at the individual, local and global levels.

## 2. Molecular Biology of Yellow Fever Virus: State of Play

In 1985, the first complete sequence of YFV was determined from complementary DNA (cDNA) clones of the 17D vaccine strain by C. Rice [44]. Thanks to the combination of these sequencing data with an NH2-terminal sequence analysis of all structural and some of the non-structural (NS) proteins, the amino acid sequence of the virus was inferred and the flavivirus gene organisation was described [45]. Despite its historical importance, only a small amount of genetic data are available for YFV species and our understanding of the virus biology is thus partly based on the data obtained for closely related flaviviruses such as dengue virus (DENV).

As for all flaviviruses, the YFV genome consists of a positive single-stranded RNA molecule ((+)ssRNA) comprising about 11,000 nucleotides (nts) with a type I cap at the 5′ terminus (m7GpppAm) [46,47] but lacking a polyA tail at the 3′ terminus [48,49]. The cap structure of flaviviral genomes is thought to be important for cap-dependent translation and to protect the genome from degradation by cellular 5′-3′exonucleases [48]. This (+)ssRNA corresponds to one large open reading frame (ORF), flanked at its 5′ and 3′ termini by untranslated regions (UTRs) that are required for RNA replication and translation [44,50,51]. Yellow fewer virus ORF spans 10,233 to 10,236 nts and encodes a polypeptide of 3411 to 3412 amino acids. The amino-terminal residues correspond to the three structural proteins while the remainder of the ORF encodes the seven NS proteins with the following organisation: 5′cap-C-prM-E-NS1-NS2A-NS2B-NS3-NS4A-2k-NS4B-NS5-3′. The 5′UTR of YFV (~110 nts) is much shorter than the 3′UTR (~400–650 nts), the size of which varies among YF strains [28,31].

### 2.1. A Highly Structured and Slowly Evolving Positive Single-Stranded RNA Genome

The cis-acting replication elements (CREs) are RNA sequence motifs and secondary structures that take part in the replication of the virus. Most of the CREs that have been identified in flaviviral genomes are located within the 5′ and 3′ UTRs. The functions ensured by CREs in the viral replication cycle have been extensively studied for DENV and West Nile virus (WNV) [52,53,54,55,56]. Several CREs are conserved across numerous flaviviral species and are thus likely to perform similar functions, as comprehensively reviewed by several authors [51,57].

#### 2.1.1. Promoters

**Figure 1:**
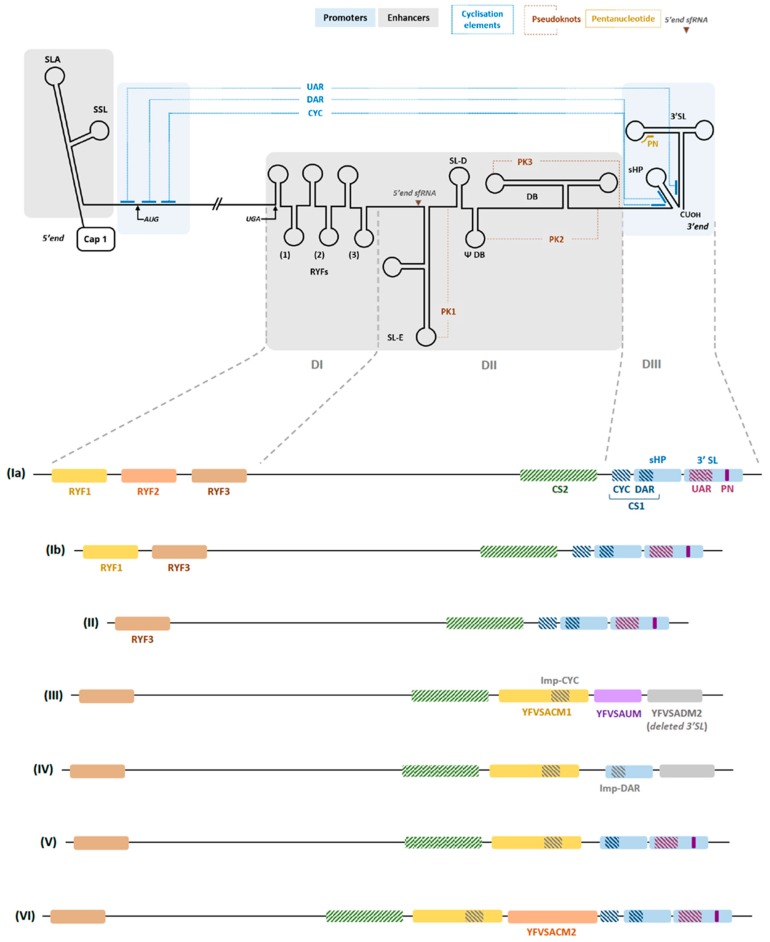
Functional sequences and secondary structures within Yellow fever virus (YFV) genome: diversity among genotypes (Modified from [57] and [87]). (Upper part) Functional sequences and secondary structures within YFV genome. Promoter and enhancer elements are highlighted in light blue and grey, respectively. At the 5′ end: Cap-1 and functional RNA structures SLA and SSL. The translation start (AUG) and stop (UGA) codons encompassing the complete coding sequence (CDS) are indicated in italics. Sequence motifs involved in viral genome cyclization are shown in blue: UAR, DAR and CYC sequences. The PN is shown in dark yellow. The 3′UTR is organized into three domains (I, II, and III). Domain I: functional RNA structures RYF1, 2 and 3 (RYFs). Domain II: functional RNA structures SL-E, SL-D, ΨDB and DB. The pseudoknot elements (PK1, PK2, and PK3) are indicated by dashed, maroon lines, and the corresponding interacting sequences, by solid lines. The 5′ end of YFV the subgenomic flavivirus RNA sequence is indicated by a triangle. Domain III: functional RNA structures sHP and 3′SL. The PN sequence “CACAG” is indicated by a dark yellow line. (Lower part) Schematic representation of the seven patterns described for the untranslated regions (UTRs) of YFV strains. (Ia) Full “native” sequence corresponding to pattern “I” corresponding to West African genotypes I and II. (Ib) Only two RYFs (1/3) are present in pattern “Ib”, which is associated with the Angolan, East and East/Central African genotypes. (II) Only one RYF (3) is present in pattern “III” (deletion YFVSADM1), it is associated with the South American II genotype. Patterns “III”, “IV”, “V” and “VI” correspond to sequences from South American I genotype. (III) Pattern “III” involves the deletion YFVSADM1 and 2 and the insertions YFVSACM1 and YFVSAUM. (IV) Pattern “IV” involves the deletions YFVSADM1 and 2 and the insertion YFVSACM1. (V) Pattern “V” involves the deletion YFVSADM1 and the insertion YFVSACM1. (VI) Pattern “VI” involves the deletion YFVSADM1 and the insertions YFVSACM1 and 2. Motifs: YFVSADM1: deletion of RYF1 and RYF2; YFVSADM2: partial deletion of 3′SL (incl. disruption of the PN); YFVSACM1: insertion disrupting the CYC sequence and including an imperfect cyclization sequence (imp-CYC); YFVSACM2: insertion upstream CYC sequence; YFVSAUM: insertion of a unique motif (YFVSAUM) disrupting the DAR sequence and the sHP structure, partly deleting the 3′SL (=YFVSADM2). Abbreviations: CS1/2: conserved sequences 1/2, CYC: cyclization motif, DAR: Downstream of AUG region motif, DB: dumbbell, imp-CYC: imperfect CYC, imp-DAR: imperfect DAR, PN: pentanucleotide sequence, RYF: imperfectly repeated sequences, YFVSAUM: South American unique motif, YFVSACM1/2: South American conserved motif, YFVSADM1/2: South American deleted motif, sHP: small hairpin structure, SL: stem-loop, SSL: side stem-loop, UAR: Upstream of AUG region.

Some CREs are crucial protagonists of the genome replication cycle and are therefore called “promoters”. During the replication of YFV genomic (+)ssRNA, the interaction between the 5′ end of the genome and the 3′UTR is critical for the recruitment and correct positioning of the NS5, a viral RNA-dependent RNA polymerase (RdRp), for the initiation of the (−)ssRNA synthesis [50,52,58,59]. This circularization mechanism is probably ubiquitous among flaviviruses [60,61,62] and involves several sequence motifs that ensure long-distance RNA-RNA interactions across the genome (see Figure 1a).

The first conserved sequence is located at the 5′ end of the YFV genome (5′CS) and its complementary sequence lies at the 3′ end (CS1). Their interaction was shown to be critical for YFV replication in cell culture [63]. The 5′CS includes the 5′ cyclisation (CYC) and downstream initiation AUG region (DAR) motifs and the CS1 involves the 3′ complementary CYC and DAR motifs (see Figure 1). The most distal promoter motif at the 5′ end is the upstream initiation AUG region (UAR) motif, which interacts through base-pairing with the 3′UAR. 5′ and 3′ UAR elements are not part of the 5′CS/CS1 motifs. The interaction between the 5′ and 3′ CYC motifs is essential to the formation and stability of the circular, replication-competent conformation of the RNA [64]. In YFV the genome, CYC motifs are 18 nts long and are found in the capsid coding sequence (nt positions 146–163) and in the 3′UTR (nt positions 10752–10765) [50]. Of note, in the 3′UTR of some Brazilian isolates from the South America I (SAI) genotype, the CYC sequence is deleted and circularization is preserved through an alternate long-range RNA-RNA interaction between the 5′ CYC and an imperfect 3′ CYC motif, as shown in Figure 1b. Finally, base pairing between the 5′ and 3′ elements of the UAR and DAR favours the initiation of the (−)ssRNA synthesis during WNV and DENV replication [64,65,66,67,68,69,70].

#### 2.1.2. Enhancers

The replication enhancer elements are not essential for viral replication in vitro but increase its efficiency. However, they may be far more important for viral replication and transmission in vivo [71,72]. At the 5′ end of the YFV genome (see Figure 1a), the stem loop A (5′ SLA) folds into a main stem loop from which emerges a smaller side stem-loop (SSL) [72,73]. This structure enables the binding and activity of the NS5 protein that ensures genome replication [58,66,74,75]. The 5′ SLA is also involved in directing the addition of the cap-1 structure at the 5′ end of the genomic RNA [47,70].

The 3′UTR is constituted of three domains (I to III), from the most proximal to the most distal (see Figure 1a) and varies importantly in length among YF strains, notably from the SAI genotype. Domains I and II include secondary structures that likely modulate the efficiency of genome replication but may not be essential to the viral replication while several elements of the 3rd domain (DIII) are crucial to YFV viability.

The first domain diverges significantly among flaviviruses and in the YFV genome, it includes imperfectly repeated sequences (RYFs) of 41 to 44 nts [76,77], the biological function of which remains to be elucidated. They include two hairpin structures that are not necessary for viral replication, either in vertebrate or invertebrate host cells [31,63,76]. In YFV species, the number of RYF varies according to the genotype (see Figure 1). Sequences from the West African genotype include three copies of the RYF (RYF1/2/3) whereas Angolan, East and Central African involve two copies (RYF1/3) [76] and those from South America I and II genotypes only contain a single—and in some cases partial—RYF (RYF3) [31]. Although the scenarios differ regarding the evolutionary history of RYFs within the ancestor of South American lineages, they converge on the view that South American genotypes of YFV evolved from West African genotypes and that this evolution involved the deletion of some RYF(s) [31,76,77].

Although the second domain (DII) is less variable across flaviviruses than the DI, YFV DII differs from that of Japanese encephalitis virus (JEV) and DENV groups. Folding analyses revealed that this domain involves two stem-loops (SLE and D), a hairpin, a dumbbell (DB) and a pseudo-DB (ψ-DB) (see Figure 1a). Sequence similarities between the linear and folded regions of DII enables the formation of pseudoknots (PK1, 2 and 3) which may increase the stability of the secondary structures [78,79]. Comparisons between wild-type and vaccinal YF strains suggest a strong association between the integrity of the SLE and virulence, as most genomes of YFV vaccine strains exhibit altered SL and PK structures [79]. The DB structure and the conserved sequence it includes are common to JEV and DENV groups and may participate in viral translation [80] and RNA synthesis [81,82]. Finally, SLE is involved in subgenomic flavivirus RNA (sfRNA) formation during YFV infection [81]. Subgenomic flavivirus RNA are found in mosquito-borne flaviviruses and result from the incomplete degradation of the viral genome by the host 5′-3′ exonuclease Xrn1 that stalls at the level of SLE [83,84]. They play an important role in virus-vector interactions, replication, cytopathology and pathogenesis [85]. It has been shown that sfRNAs interfere with the cellular RNA decay machinery and the interferon type I pathway, but they may act through other mechanisms, as comprehensively reviewed elsewhere [86]. Finally, insertions of specific sequences, now referred to as the South American conserved motifs 1 and 2 (SACM1 and 2), can be found in the DII of some genomes from the SAI genotype, in a lineage-independent manner [31,87]. Most likely, these insertions were generated through the duplication of a conserved stem-loop structure. The SACM1 insertion disrupts the CYC sequence but includes an imperfect CYC sequence (imp-CYC) that ensures the circularization of the genome through an alternate interaction with the 5′ CYC sequence (see Figure 1b) [87].

Domain III is highly similar between flaviviruses and contains a small hairpin (sHP), a large terminal stem-loop (3′SL) structure, as well as the 3′ cyclization motifs (CYC, DAR and UAR). The 3′SL was found to be essential for YFV replication *in cellulo* [63]. Furthermore, studies on WNV, DENV and Kunjin (KUNV) virus demonstrated that both sHP and 3′SL are necessary for the completion of the viral cycle and may establish interactions with viral and non-viral proteic processes [52,60,88,89,90,91,92,93,94,95,96,97,98]. However, a deletion, the South American deleted motif 2 (SADM2), has been reported in some Brazilian strains from genotype SAI, and has been predicted to—at least partially—disrupt the sHP and 3′SL structures [87]. Hence, as viruses with minimal versions of the sHP/3′SL are viable, the dependence of YFV cycle on the sHP and 3′SL structures may not be strict [87]. Finally, a conserved pentanucleotide (PN) sequence 5′-CACAG-3′ is located within the loop of the 3′SL structure, the most distal part of the 3′UTR. The conservation of this PN sequence was shown to be essential to KUNV and WNV replication but for YFV, the dependence is likely to be significantly looser and the role of this PN sequence within the viral replication cycle remains to be determined [93,99]. In vitro experiments demonstrated that YFV 17D-derived mutants with altered PN sequences were viable under experimental conditions [100]. As the SADM2 deletion that has been identified in Brazilian isolates encompasses the PN sequence [87], the latter is not likely to be involved in RNA-RNA interactions for such a mechanism would require a much tighter conservation. Rather, the PN sequence could be part of a host or viral protein binding site [100].

Overall, the biological importance of duplications and deletions within the 3′UTR of YFV strains (RYFs, YFVSACM1/2, YFVSADM2) remains to be elucidated and should provide most valuable insights regarding the regulatory role of the RNA secondary structures and motifs they involve. There are several experimental demonstrations that mutations within flavivirus 3′UTRs have a differential impact depending on the host system (insect or mammalian) (e.g., [92,101,102]). Villordo and colleagues have comprehensively reviewed the present knowledge of the relationships between secondary structure RNA elements and host specialization in flaviviruses [101]. Elucidating the role of the structured elements of the YFV genome in the interactions between the virus and its hosts/vectors will be most useful to understand how virus-vector-host networks can shape viral evolution and may open new research avenues for tuning host-vector preference within flaviviral genomes.

#### 2.1.3. A Genome That Evolves Relatively Slowly

RNA viruses typically have higher mutation rates than DNA viruses because their replication involves low-fidelity viral RdRps that frequently do not have proofreading activity. Hence, while for most DNA viruses, the mutation rate ranges between 10^−8^ and 10^−6^ substitutions/site/generation, for most RNA viruses it is much higher, with 10^−5^ to 10^−3^ substitutions/site/generation [103]. Since the nucleotide substitution rate (or evolutionary rate) depends on the mutation rate, the replication rate and the subsequent selection, an elevated mutation rate is not necessarily indicative of fast evolution. As dual-host pathogens, arboviruses and, notably, flaviviruses exhibit low evolutionary rates (<10^−3^ substitutions/site/year) [104] when compared with other RNA viruses such as avian influenza A virus, or the human immunodeficiency virus (HIV) (2 to 8 × 10^−3^ and 3 to 8 × 10^−3^ substitutions/site/year, respectively) [105,106,107]. Yellow fever virus appears to have a relatively low evolutionary rate, with 2 to 5 × 10^−4^ substitutions/site/year, as reported by several studies of the prM/E junction coding sequence [87,108,109,110]. In terms of evolutionary behaviour, marked differences have been observed between YFV and other flaviviruses with close ecological features such as DENV, which replicates in similar hosts and shares common vector species but exhibits evolutionary rates ranging from 7 to 9 × 10^−4^ substitutions/site/year according to the serotype [108,111]. Several hypotheses have been put forward by Sall and colleagues to explain the differences in evolutionary dynamics between YFV and DENV. They suggest that a preponderant role of transovarial transmission (TOT) within mosquitoes in the maintenance of the virus could be implicated as such a mechanism, where the virus may remain quiescent in mosquito eggs for many months, would decrease the replication rate of the virus [108]. As detailed in the first part of this review, several elements indicate that the TOT mechanism is not likely to play a significant role in YFV maintenance, neither in Africa nor in South America [112]. Notably, the rates of infection of the progeny observed under laboratory conditions are too low to enable the long-term survival of the virus through a TOT mechanism [113]. Hence, a TOT mode of survival is not likely to be preponderant in YF maintenance and would thus have a minor impact on the evolutionary rate of the virus. Finally, YFV and DENV differ importantly in terms of epidemiology, as DENV can establish sustained chains of transmission in humans while YFV does not (see first part of this review [112]). These distinct epidemiological trends could be the source of differences in evolutionary dynamics as these can be modulated according to the dynamics of transmission and between intra and inter-epidemic periods (e.g., [114]). Further investigations and comparisons using larger sequence datasets are thus needed to identify the possible mechanisms resulting in the apparently slow evolution of YFV. These could be intrinsic to the virus (mode of replication, polymerase fidelity) or extrinsic (ecology, epidemiology, immune response). Finally, in addition to mutations, recombination between viruses can further participate in sequence diversity within a given species. Recombination has been reported for some tick and mosquito-borne flaviviruses [115,116,117,118,119,120,121] but this has never been the case with YFV and experimental data further suggest that recombination between YFV strains is unlikely [122]. However, the mechanism by which the insertions and deletions identified within YFV 3′UTR have been generated remains to be described and could imply intra or inter-molecular reactions close to a recombination mechanism [31].

Originally defined on a serological basis, the YFV species corresponds to a single serotype and includes seven genotypes (West I, West II, East/Central and East Africa, Angola and South America I and II). The first genotypes were identified as topotypes, using RNAase T1 oligonucleotide fingerprinting techniques [123,124]. Genotype definition has progressively emerged and was formally outlined by Mutebi and colleagues in 2001 [125]. They defined genotypes as “distinct lineages which differ by greater than 9% at the nucleotide sequence level”, a criterion that had previously been used in several studies including their own [125,126,127]. It is commonly considered that the evolutionary rate is homogeneous across YFV genomes, with no significant variation across specific coding regions (e.g., structural versus NS proteins), as comprehensively detailed by Beasley and colleagues [28]. Hence, in the 1990s and 2000s, YFV molecular phylogenies have been performed using a large variety of sequences, including structural and NS protein coding sequences, as well as UTRs [31,76,77,125,127,128,129,130]. Over the last decade, YFV molecular phylogenies have primarily been performed on the complete genome of the virus or on a partial sequence of ca. 670 nt, called the “prM/E junction” [125], that encompasses 108 to 334 of the 3′ nts of the prM coding sequence, the full-length M protein coding sequence and the 337 5′ nts of the E coding sequence [30,87,109,110,131,132,133,134,135,136,137,138,139,140]. However, the NS5 coding sequence and the 3′UTR are still used on some occasions [137,141]. As detailed in the first section of this review, there is a strong association between the phylogenetic and the geographic clustering of YFV strains so genotypes correspond to specific geographic areas that rarely overlap [112]. This is suggestive of a predominant role of in situ evolution in shaping YFV diversity, consistent with the maintenance of YFV in a primarily “sylvatic” mode of transmission [142]. As comprehensively reviewed by Beasley and colleagues, and as it may appear in the above paragraphs, the association between specific genotypes or lineages and phenotypic features such as virulence or adaptation to specific vector(s) and/or host(s) is severely understudied [28] and much remains to be learned regarding the biological significance and determinants of YFV genetic diversity.

### 2.2. Structure and Replication of the Viral Particle

Yellow fever virus is an enveloped virus that replicates in the cell cytoplasm throughout a cycle which is usually defined in seven contiguous stages: (i) attachment to the cell surface, (ii) internalisation into the host cell, (iii) fusion and transfer of the viral RNA genome into the cytoplasm, (iv) translation of the viral proteins, (v) replication of the genomic RNA, (vi) assembly and maturation of the virions, and (vii) release of progeny viruses from the cell [143,144]. Most knowledge of the YFV replication cycle arose from studies achieved with other flaviviruses and the YFV 17D strain. As the flavivirus replication cycle has been described elsewhere [144,145,146], this section will be limited to a brief overview of the respective roles of YFV proteins within the YFV life cycle. While only the structural proteins become part of the mature infectious virion, the NS proteins play multiple roles in polyprotein processing, viral RNA synthesis, and virus morphogenesis.

Yellow fever virus is a small, enveloped virus of 50 nm diameter. The capsid (C), the membrane (prM/M) and the envelope (E) are the three structural proteins of YFV. The virion structure involves a nucleocapsid core (NC), surrounded by a host-derived lipid bilayer with an outer glycoprotein shell composed of two glycoproteins, M and E, of which 180 copies are assembled following an icosahedral symmetry [147].

The capsid is involved in genomic RNA packaging and in the formation of the NC [44,148,149]. In addition, it was recently shown that YFV C protein plays a role in inhibiting RNA silencing in *A*. *aegypti* mosquitoes by binding long dsRNAs with high affinity, thereby impeding their efficient processing by the Dicer protein [149]. The membrane protein exists under both an immature (prM) and a mature (M) form and may act as a chaperone to ensure the proper folding and assembly of the E protein [150,151]. At the surface of immature virions, prM proteins form heterodimers together with E proteins, giving the viral particles a spiky aspect [147] and preventing the adventitious fusion of the virus during egress [152]. Upon maturation, prM and E proteins successively reorganise as (prM/E) and finally as E homodimers giving a smoother aspect to the viral particles. However, most virions retain an important degree of structural heterogeneity [153] due to both incomplete furine cleavage of the prM protein [154,155,156] and the dynamic “breathing” behaviour of the metastable E dimers [157,158].

The E protein mediates the entry of the virus into the cell and interacts with cellular receptor molecules at the cell surface [143]. First, low-affinity interactions with a range of surface molecules such as heparan sulphate [24,159] and subsequently through high-affinity interactions with specific—and yet unknown—receptor molecules such as integrins, as proposed in the case of YFV 17D [160]. After recognition and attachment, the virus enters the host cell by clathrin-dependent endocytosis. The low pH environment inside the endosomes triggers a rearrangement of the E proteins in fusion-active homotrimers that facilitate fusion by bringing viral and cell membranes in close proximity [161,162,163,164,165] to form a fusion pore through which the nucleocapsid is released into the cytoplasm [166,167]. Of note, the vaccine strain YFV 17D has recently been reported to enter the cell through a clathrin-independent pathway. This switch in entry mechanism relies essentially on the 12 mutations differentiating YFV 17D E protein from that of its parental (Asibi) strain [23]. Other alterations of YFV E protein have been reported to impact viral tropism and virulence [23,27,35,168,169,170,171].

After its dissociation from the capsid protein through a still undefined uncoating mechanism [172], the viral genomic RNA is translated and replicated in the cytoplasmic compartment, in close association to the endoplasmic reticulum membranes, as a prelude to viral particle assembly. The translation gives rise to the polyprotein precursor which is glycosylated by cellular glycosyltransferases and finally cleaved, post- and co-translationally, by host and viral proteases, into three structural and seven NS proteins along with the 2K peptide. The virus-encoded serine protease (NS3-NS2B complex), ensures the cleavage between NS2A/NS2B, NS2B/NS3, NS3/NS4A and NS4B/NS5 [173,174,175]. The 2K peptide is thought to act as a signal sequence and is removed after cleavage [176].

NS5 and NS3 are major elements of the replication complex and achieve the synthesis and capping of the viral genomic progeny (flaviviral RNA replication is comprehensively reviewed in [177]). Genome replication takes place in vesicle packets that emerge through ER membrane rearrangements, a mechanism that may be induced by the NS4A protein [178,179]. During genome replication, regions of the transmembrane NS2B, NS4A, and NS4B proteins interact with the NS3 and anchor the replication complex to the ER membrane. The NS1 protein [180,181] and cellular factors may also be involved in the replication step [182], but their roles have not yet been defined. The viral genomic progeny are then used for further translation or associated with the structural proteins before being incorporated into immature virions. In addition to their role in the replication process, the NS1 protein takes part in the immune evasion of the virus (reviewed in [183]), the NS4A and NS4B are allosteric cofactors for the NS3 helicase domain [184,185] and the NS4B blocks interferon α/β signalling [186].

Immature, fusion-incompetent, viral particles are first assembled through the combination of newly synthetized viral proteins and nucleic acids [187] through non-specific, electrostatic, C-RNA interactions facilitated by the close association of replication and genome encapsidation [188,189]. Immature virions acquire a host-derived lipid envelope by budding into the lumen of the endoplasmic reticulum (ER) [190] and progress through protease- and pH-dependent maturation while they transit through the trans-Golgi network to the cell surface [152]. This maturation involves the reorganization of the E proteins and the cleavage of the prM protein by cellular endoproteinase furin proteins in to the “pr” peptide and the M protein [191,192], a mechanism which is tightly constrained by calcium concentration [193]. Once cleaved, the N-terminal pr fragment remains virion-associated until it is exocytosed, when it is then shed from the virion during mature viral particle release [194]. In this metastable structural state, virions are “fusion-competent” (i.e., able to undergo the low-pH triggered fusion events during the upcoming stage of cell entry) [195].

Flavivirus replication is considered to be primarily cytoplasmic however, some flaviviral proteins, namely the C, NS4B and NS5, can translocate to the nucleus, a mechanism that may participate in replication and/or immune suppression. The current understanding of the modalities and function(s) of the nuclear localisation of flaviviral effectors during the viral replication cycle is limited for flaviviruses in general and for YFV in particular, as comprehensively reviewed by Lopez-Denman and Jason M. Mackenzie [196]. Although the viral cycle of YFV—and other flaviviruses—has been partly elucidated, many gaps remain in our understanding of the mechanisms and of the viral/host factors that are involved in YFV replication. Improving our grasp on the molecular biology of the virus will pinpoint specific steps and components of the viral life cycle that could be targeted to develop safe and efficient antiviral strategies against YFV and possibly, other flaviviruses.

## 3. How to Mitigate and Manage YFV Infections?

### 3.1. Virus Tracking: Diagnostic Tools Inventory

The recent YFV outbreaks in Latin America [197,198] and Africa [199] demonstrated the need for reliable YFV diagnostics as a part of global YFV control. Outbreak management is largely dependent on rapid diagnosis of cases to establish appropriate mitigation measures (e.g., medical care, emergency vaccination, vector control). In addition, efficient diagnostic tools are necessary to identify the areas where the virus circulates and, on this basis, implement adequate long-term immunisation plans. The World Health Organization (WHO) estimates that YFV cases are still massively under-reported, with a true number of cases estimated to be 10 to 250 times those now being reported. Important reasons for under-reporting are asymptomatic infections and mild infections with nonspecific symptoms that are not identified by differential diagnostics [200]. To improve case-reporting, the WHO recommends that every at-risk country ensures that YF blood tests can be performed in at least one national laboratory [201]. In the past, virus isolation was a common tool in arbovirus laboratory diagnostics. Nowadays, time-saving molecular and serological methods provide the basis for arbovirus diagnostics [202]. Nonetheless, sensitivity and specificity of these methods are constant challenges to virological diagnostics and on some occasions, virologists and clinicians need to accept that unambiguous diagnoses cannot be achieved despite the usage of multiple tests. In the following, we provide details on the different diagnostic methods, discuss official recommendations and new trends, and point at gaps in our current knowledge of YFV laboratory diagnostics.

#### 3.1.1. Molecular YFV Diagnostics

Until the beginning of the 21st century, molecular YFV diagnostics was achieved partly by conventional reverse transcription PCR (RT-PCR) [203,204,205]. Subsequently, YFV-specific reverse transcription loop-mediated isothermal amplification (RT-LAMP) [206,207], real-time recombinase polymerase amplification (RT-RPA) [208] and real-time RT-PCR assays using either intercalant dyes such as SYBR Green I [209,210] or TaqMan (hydrolysis) probes [211,212,213,214,215,216,217,218,219,220] were developed. Very recently, a Specific High Sensitivity Enzymatic Reporter UnLOCKing (SHERLOCK) was developed for DENV and Zika virus (ZIKV) detection by using the RNA-targeting CRISPR-associated enzyme Cas13 [221,222]. This innovative method could be adapted for YFV detection, but its robustness in clinical use remains to be demonstrated. Nowadays, TaqMan assays are most commonly used due to their specificity and ease of use, notably because the probe format is available across western and resource-limited regions at affordable prices. However, molecular protocols apply to a short timeframe of acute infection due to short-lived viremia in arbovirus infections [223,224]. Although potentially extended in severe cases [225,226,227], viraemia is frequently short and peaks around 2–3 days following YFV infection and in most cases, the viral RNA can only be detected in the blood for 3–6 days post-infection. In addition, viral loads are often low and vary significantly, ranging between 10^2^ and 10^7^ genome copies per mL [228,229,230,231,232,233,234]. As for other flaviviruses [235,236], YFV RNA can be detected in urine and semen but unfortunately, we lack reliable data on the frequency of detection of the virus in urine/semen samples. The closely related ZIKV is detected in urine with a 50–95% frequency [237,238,239,240] and in semen in up to 33% [236] of confirmed male cases. Noteworthy, prolonged detection of ZIKV RNA was reported in urine/semen as compared to serum [237,238,240,241], with semen being tested positive up to six months after the onset of symptoms [242]. On this basis, it has been suggested that testing urine might improve molecular ZIKV detection [237,238]. Nonetheless, efficiency may vary depending on cohorts and with regard to the type of test that is used [223]. Overall, most information on YFV viraemia comes from single case reports or vaccine studies and more reliable data from the field are urgently needed. Notably, it would be worthwhile determining if higher YFV loads or prolonged viraemia correlate with disease severity as reported for other arboviruses [243]. Due to the short time-span of YFV presence in blood, official WHO [244] and Pan American Health Organization (PAHO) [245] guidelines advise molecular YFV testing for up to 6–10 days following the onset of symptoms, followed and complemented by serological methods. The complexity of molecular YFV diagnostic was also revealed by external quality assessments (EQA) published in 2012 and very recently in 2018 [246]. In 2012, among participating laboratories from Europe, the Americas, Middle-East, Asia and Africa, 84% needed to improve their testing procedures. The major problems that were reported were lack of sensitivity relative to wild-type viruses and lack of specificity [247]. Within the 2018 EQA, 47% of the participants revealed major problems [246].

#### 3.1.2. Serological YFV Diagnostics

Serological flavivirus diagnostics are commonly recommended from day six post-infection onwards [244,245] and are usually based on the detection of specific immunoglobulin M (IgM) or immunoglobulin G (IgG) antibodies. IgMs are developed a few days after infection and can be detected for up to six months [248] whereas IgGs are developed during convalescence but can usually be detected for decades [249]. In both cases, seroconversion using paired sera can confirm acute infections, but such samples are not always available from remote areas. The most commonly used biological specimens for YFV serology are serum and plasma. Although other specimens, including cerebrospinal fluid (CSF), are used for tick-borne encephalitis virus (TBEV) serology [250], YFV IgM detection in CSF was only reported for vaccine-associated adverse events (YF-VAAE) [251,252].

A variety of enzyme-linked immunosorbent assays (ELISA) [253,254], indirect immunofluorescence assays (IFA) [255,256], microsphere immunoassays (MIA) [257] or the plaque reduction neutralization test (PRNT) [258,259,260] procedures are operable for YFV serology. PRNT_50_ and PRNT_90_ are used to detect neutralizing antibodies [259,261,262,263] and are considered the gold standard for arbovirus serology [244,264]. However, largely for technical reasons, including the need for biosafety level 3 (BSL-3) laboratory facilities (or in some Asian countries, BSL-4), PRNT is used less frequently than other methods. Recently, a non-infectious virus-like particle (VLP)-based YFV PRNT was developed, partly to overcome these problems, that enables final analysis by flow cytometry [265].

Finally, while the PRNT still remains an extremely valuable diagnostic tool, flavivirus serology can be challenged at different levels. First, the kinetics of antibody responses can be influenced by former flavivirus infections [266] or pregnancy [267] and might vary greatly between individuals [268]. Second, serological diagnostics are hampered by broad cross-reactive anti-flaviviral antibodies that may cause false-positive test results [253,268,269]. Due to the complexity of arbovirus serology, precise guidelines for serologic YFV diagnostics were provided by the WHO. Suspected YFV cases are confirmed serologically by (i) the detection of YFV-specific IgM antibodies in the absence of IgM against DENV, WNV and ZIKV without recent YFV vaccination history, (ii) by a fourfold increase in YFV IgM/IgG titres between acute and convalescent blood specimens, (iii) by the presence of YFV neutralizing antibodies without YFV vaccination history, or (iv) by detection of YFV antigens using an immunoassay without recent YFV vaccination history [244]. Unfortunately, there is only limited knowledge concerning the performance of diagnostic laboratories ensuring the quality of YFV serology. The latest EQA on YFV diagnostics was published in 2012 and revealed problems of specificity and, more importantly, sensitivity, as almost 50% of the participants failed to detect YFV 17D IgM [247]. These problems are not unique to YFV diagnostics, as reported for other arboviruses such as DENV [270] or WNV [271], but are of concern given the medical relevance of YFV. New EQAs are suitable to assess the actual capacity of diagnostic laboratories and might be the key to optimizing YFV diagnostics.

#### 3.1.3. Changing YFV Diagnostics in Times of Mass Vaccination Campaigns

Recently, the demand for new molecular YFV diagnostics became evident. In December 2016, Brazil reported the largest YFV outbreak for decades and launched an extensive vaccination programme. During the past 80 years, live attenuated YFV 17D and YFV 17DD vaccines have proven to be safe and efficacious for use in humans >6 months of age and many millions of doses have been administered globally. However, even using the existing carefully regulated live-attenuated YFV vaccines, approximately 0.4/100,000 cases of YF-VAAE are estimated to occur [272] and mass YFV vaccination thus raises the need to differentiate between YF-VAAE and wild-type infections [273]. Discrimination of vaccine and wild-type YFV strains is hindered by low genetic divergence within YFV species. This is particularly important in the case of Western African genotypes, which are closely related to the Asibi strain, the parental strain of modern YFV vaccines. Accordingly, new real-time RT-PCR assays discriminating between wild-type and vaccine strains [218] or specifically detecting vaccinal strains [219] were recently published. Molecular YFV diagnostics are further challenged by the application of the new chimeric, live- attenuated DENV vaccine Dengvaxia^®^ in countries that are endemic for YFV such as Brazil [274,275,276]. This vaccine is based on a YFV 17D-backbone expressing the prM and E regions of the four DENV serotypes [277]. Most RT-PCR assays listed above would falsely detect this vaccine as a wild-type YFV hence new, discriminant assays are needed. Noteworthy, DENV-based DENV vaccines are currently in phase three clinical trials [278]. Their application should overcome problems in differentiation between DENV vaccination and YFV infection.

#### 3.1.4. Limited Information about Genetic YFV Diversity

**Figure 2:**
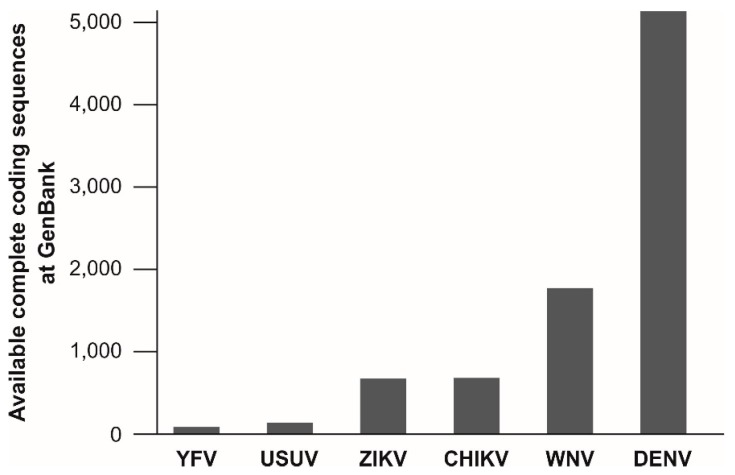
Number of available complete coding sequences at GenBank for selected arboviruses. Sequences were accessed on 15 May 2018. Viruses are ordered by the number of available sequences. Chikungunya virus was included for comparison. CHIKV, Chikungunya virus; DENV, dengue virus; USUV, Usutu virus; WNV, West Nile virus; YFV, Yellow fever virus; ZIKV, Zika virus. YFV, *n* = 88; USUV, *n* = 142; ZIKV, *n* = 677; CHIKV, *n* = 684; WNV, *n* = 1778; DENV, *n* = 5145. For YFV, 49 sequences originate from the Americas (including 40 from Brazil), 28 from Africa and 11 from Asia (including five cases imported from Africa).

The target regions of published molecular assays for YFV detection are not evenly spread across the viral genome. Of at least 20 published assays for molecular YFV diagnostics, six target the 5′UTR, five the NS5 coding region and four the NS1 coding region. Remarkably, the information about the genetic variation of YFV is relatively small and is limiting reliable oligonucleotide design for YFV detection. As of 15 May, only 88 complete coding sequences of YFV isolates are available at GenBank, NCBI. This number is surprisingly low given the long time-span since the discovery of YFV and given its medical relevance. In comparison, the genetic information available for other arboviruses is considerably greater, including for the bird-associated Usutu virus (USUV), that causes only sporadic human infections [279] (Figure 2). This underrepresentation of YFV in terms of genetic data might be a major problem for public health since existing real-time RT-PCR assays are designed based on limited knowledge about genetic YFV diversity and nucleotide mismatches at oligonucleotide binding sites may affect test performance [223,280]. Despite the relatively low evolutionary rate of YFV [28,108,133], larger YFV genomic datasets are urgently required to ensure reliability of molecular YFV diagnostics.

### 3.2. Infection Prevention in Endemic and At-Risk Areas

#### 3.2.1. Vaccination Policies

YFV vaccination is based on the use of the attenuated YFV 17D strain, which was originally elaborated by M. Theiler in 1935 [38]. This live-attenuated vaccine is commonly recognized as one of the most effective ever created and vaccines are still being manufactured using its substrains, YFV 17D-204 and YFV 17DD, as seeds [2]. Although differences in antigenicity between YF strains have been reported on some occasions [281,282,283,284,285,286,287], the view that the YFV species corresponds to a single serotype is commonly accepted and it is considered that immunisation using either YFV 17D-204 or YFV 17DD-derived vaccines confers protection against all circulating YFV strains. YFV vaccine and vaccination have been comprehensively reviewed by several authors over the past decade [2,145,272,288,289]. For the sake of parsimony, we will thus focus on the major issues regarding YFV vaccination practices.

Currently, population immunisation is ensured through both routine and emergency vaccination efforts.

**Figure 3:**
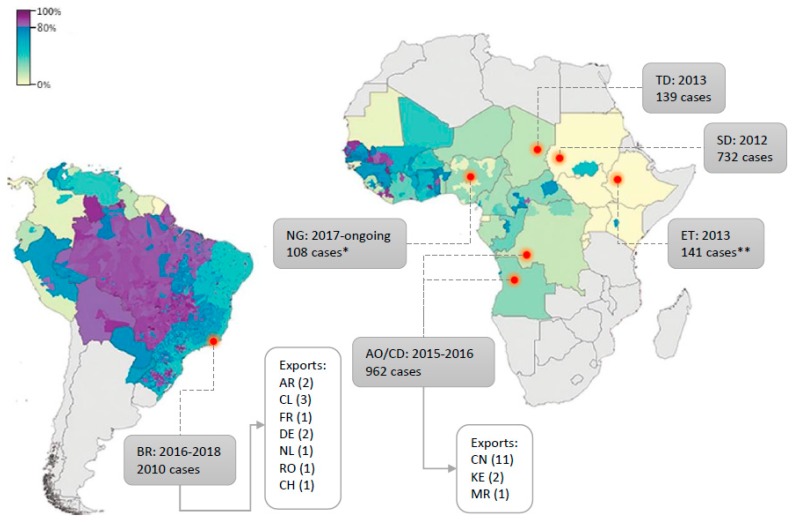
Yellow fever (YF) outbreaks since 2010 with regard to vaccination coverage at the time (modified from [299]). Estimated proportion of the population (across all ages, in %) to have ever received a YF vaccine at the beginning of 2010 for countries at risk of YFV transmission, based on the untargeted, unbiased vaccination-targeting scenario [299]. Countries where YFV outbreaks occurred since 2010 are indicated by a red dot. Outbreak time-span and total number of cases are detailed in grey boxes. Export cases are detailed when applicable. All country names’ abbreviations are defined according to international ISO country codes. * Presumptive positive cases ** reported cases.

Routine vaccination campaigns are common in endemic areas but vary in width and with regard to the target population according to the regions of the world. Notably, the low vaccination coverage in the endemic areas of East Africa which historically recorded weaker YF activity is likely to have contributed to the advent of important outbreaks in these regions [145] as in 2010 in Uganda [290,291], in 2012–2013 in Ethiopia and Sudan [292], and more recently in Central Africa, with several thousand suspected cases reported between 2016 and 2017 in Angola and DRC [199,293]. In addition, the recent outbreak in Brazil reached areas with no vaccination recommendation hence, with a potentially low proportion of immunised inhabitants [198] and similarly, the ongoing outbreak in Nigeria takes place in an area with presumably low vaccination coverage [43]. A map recapitulating YFV outbreaks over past decades, with estimated vaccination coverage, can be found in Figure 3. Such events are blatant indications that routine vaccination should be taken to a larger scale, regarding both the target populations and the areas encompassed.

Emergency vaccination efforts can be mounted during outbreaks, notably when they occur outside endemic areas or reach uncommonly large magnitude, as illustrated by the large campaign that was established to contain the 2016–2018 outbreak in Brazil [198]. In 2016–2017, the virus reached the South-Eastern part of the country, notably the metropolitan areas of Belo Horizonte, Rio de Janeiro and Sao Paulo, which are densely populated and include numerous localities where the vaccine was not recommended. This immunisation initiative was unprecedented in that it aimed to reach an extremely large population of ca. 26 million people [42] notably thanks to the adoption of dose-fractioning strategies [294,295]. The first large use of the dose sparing approach dates back to August 2016, in Kinshasa, when more than seven million people, including children from two years of age, were immunised using 1/5 of the YFV 17DD vaccine to contain the 2015–2016 outbreak in a context of vaccine shortage [296]. This procedure was established again in 2017–2018 in Brazil, and more than seven million people have been vaccinated using 1/5 of the standard dose as of epidemiological week 18 (1st week of May) [42,297].

The WHO currently recommends that fractional YF vaccine dosing should be used as a short-term response to outbreaks during periods of shortage of full-dose YF vaccine. In practice, the lowest dose that can be used is of 1000 IU and should be administered subcutaneously or intramuscularly. Finally, the WHO recommends the use of the YF-17DD vaccine for dose-sparing procedures, as the available immunogenicity and safety data were obtained for this specific vaccinal strain [298].

Recent results from a dose-response study in which young adult males were immunised using dose-tapered YFV 17DD vaccine [295] demonstrated that all seroconverters remained seropositive eight years later, for doses ranging from 31 to 27,476 UI [297]. Such insights are crucial to evaluate the necessity and timing for re-vaccinating the increasingly large populations that have been immunised using fractionated doses over the past years in DRC and Brazil (>12 million people). Further investigations are needed, as the dose-response study included exclusively young adult males while the vaccine is recommended for individuals of nine months of age or older. Indeed, differences in immune response among age groups were reported during the dose-fractioning campaign in Kinshasa [296], so there is a risk that the duration of immunity may be reduced in young children and adults over 50 years [297].

Intradermal administration of the vaccine has been proposed as an additional means of reducing the dose necessary for effective immunisation against YFV [29,298,300,301,302,303]. In the past, this approach has proved to be fully efficient in the context of vaccination against variola, tuberculosis and more recently, influenza virus [294,301]. This procedure offers the advantage of mimicking the natural infection better than intramuscular and subcutaneous routes and is believed to elicit cell-mediated immunity [298,301,304]. Overall, intradermal immunisation appears to be a relevant strategy for ensuring efficient YF vaccination in the context of limited vaccine stocks and there is first evidence of a similar effectiveness between the intradermal administration of a low dose YFV 17D vaccine (Stamaril) and standard vaccination [303]. Further investigations are needed to provide full evidence of superior or equal efficacy of the use of the intradermal immunisation when compared to conventional routes, for all clinically available vaccines, to enable this strategy to be taken to the next level.

#### 3.2.2. Vector-Control Plans: Past, Present and Future Strategies

Despite the existence of an effective vaccine for YFV, preventing and reducing (during epidemics) viral transmission also depends on controlling the mosquito vectors. The sylvatic transmission of YFV has no possibility of vector control because of the multiplicity and/or the inaccessibility of natural breeding sites (mainly tree holes) used by sylvatic mosquito species in Africa or in the Americas (see the first part of the review for more details). Due to potential adverse effects of insecticides on non-targeted organisms, the use of insecticides against both immature and adult stages is also inapplicable in natural ecosystems. The only way to prevent the contact between human and sylvatic vectors remains limited to personal protection using topical repellents or insecticide-treated clothes.

The urban transmission of YF is mainly supported by *A*. *aegypti*, which also ensures the epidemic transmission of emerging *Aedes*-borne viruses that pose growing global threats. All vector control programmes developed during the past decades to control dengue, chikungunya or Zika epidemics and *A*. *aegypti* (and *Aedes albopictus* to a smaller extent), are transposable to YFV. Top-down vertical *Aedes* control programmes have been successfully applied to control YF in the Americas (1900–60s) and more recently dengue, in Singapore (1970–80s) and Cuba (1980–90s) [305,306,307]. However, these large, paramilitary and vertically structured programmes, that include thousands of inspectors and other public health staff with enforcing laws that prohibit owners from allowing mosquito production in their dwellings (top-down campaigns) have become impossible to implement in the modern era. Nowadays, community participation programmes (bottom-up approaches) such as the use of communication for behavioural impact [308] in cities, could be efficient to control *Aedes* [308,309]. To curtail YFV transmission in urban environments, Integrated Vector Control Management (IVM) approaches can combine several methods including personal protection, larval (source reduction, larviciding and biological control) and adult (adulticiding) vector control measures [310].

Among relevant control methods, those targeting adult mosquitoes with insecticides can be deployed as an ‘emergency’ measure for preventing transmission as peri-domestic treatments carried out in and around households where human infection has been reported. Adulticiding can be done with different methodologies, space spraying versus residual spraying, indoor versus outdoor, house-to-house application using portable equipment versus vehicle-mounted fogging and cold versus thermal fogging. Insecticides can be useful to curtail transmission when applied properly but they have several constraints as insecticide resistance, low societal acceptability, environmental impact and potential impact on non-target organisms. In relation to personal protection measures, the use of insecticide-treated materials and house screening has been shown to be effective [310]. Topical repellents can be useful for individual protection and can be distributed during epidemics. Larval control, such as environmental management, source reduction, larviciding or biological control is more effective when it is consistent and routinely used rather than a haphazard emergency response. Source reduction should primarily target artificial containers in private and public spaces and may also include some natural containers such as bamboos and bromeliads, that can also harbor *Aedes* larvae. Community-based campaigns of source reduction have proved to be efficient for controlling disease transmission [309]. Several larvicides (Bti, temephos, pyryproxifen or diflubenzuron) and biological control methods (fish or copepods) can be used for the treatment of large and permanent breeding sites such as drinking water containers. Other control methods, such as sterile and transgenic mosquitoes, mass trapping or Wolbachia-based strategies are being developed but there is insufficient evidence to recommend their application at present [311].

These control measures should be applied in an integrated manner, with community mobilization and adequate inter-sectoral collaboration. Epidemiological and entomological surveillance may guide their implementation in areas with high transmission risk (e.g., neighbourhoods). Integrated Vector Control Management must be conducted in a precise, sustainable and proactive manner, targeting both larvae and adults, and if possible, in combination with vaccination campaigns increasing the effectiveness for controlling and reducing YFV transmission.

### 3.3. Patient Care: Perspectives for Treatments against Yellow Fever Virus Infection

To date, no specific drug is available for YF treatment, which remains limited to symptomatic and supportive care. Although different molecules have shown in vitro and/or in vivo activity against YFV, none is available clinically. The specifications for antiviral compounds depend on the purpose of their intended use. In the case of an outbreak or VAAE, an ideal curative anti-YFV molecule should be effective if administered directly after the onset of symptoms associated with YF disease. On the other hand, for a preventive use (e.g., in travellers), requirements may be looser in terms of efficiency, as the drug can be administrated ahead of the infection. However, higher standards are required in terms of safety, for the benefit/risk ratio will depend on the probability of infection. Efficient antiviral drugs can target effectors involved in the viral cycle or in pathogenesis, whether viral or cellular.

Both structural and NS proteins of YFV have been proposed as targets for antiviral drug design.

Targeting the structural proteins and notably the envelope of YFV is used to interfere with the early stages of the viral cycle by blocking viral entry or through neutralization of virus infectivity. Several candidate molecules have been identified based on modelling and in silico screening studies, some of which have proven effective in inhibiting YFV replication in vitro [312,313,314,315,316,317,318,319]. So far, passive immunisation using sera from vaccinated hamsters is the only procedure that has proved to ensure effective YFV neutralisation in vivo [320]. Finally, another option is to suppress E protein expression using RNA interference. Thanks to this approach, adult BALB/C mice were protected against YFV following intra-cranial injection with the YFV 17DD strain [321].

Inhibition of the activity of YFV NS proteins has been widely investigated in the last decade, with compounds targeting the NS1 protein [321], the NS2B-NS3 complex [322], the NS3 [322,323], the NS4B [324,325] and the NS5 proteins [326,327,328,329]. Among all the types of molecule that have been tested for anti-polymerase activity, some exhibited significant efficiency in vivo and notably, compounds corresponding to the group of nucleoside analogues. The pyrazine carboxamide T-1106 has shown activity against YFV in the hamster model [330] and treatment with the T-705 (Favipiravir), an FDA-approved chemically related compound, significantly improved disease parameters in YFV-infected hamsters when beginning administration up to three days post-infection [331]. Of note, T-705 in vitro activity against YFV was synergistically enhanced when combined with the ER α-glucosidase inhibitor IHVR-19029 [332]. Finally, a novel adenosine analogue, the pyridine carboxamide BCX4430, offered complete protection from mortality with significant improvement of other disease parameters in a hamster model, and remained effective even when treatment was initiated at the peak of viral replication (i.e., four days post infection) [333]. Overall, targeting the viral polymerase (NS5) presents several advantages. First, the absence of a human host equivalent to this enzyme makes likely that inhibition of the NS5 RdRp activity is only associated with low levels of toxicity [334]. Second, because of the uniqueness and importance of the NS5 polymerase activity, there is a high degree of structural conservation of the NS5 polymerase domain among *Flaviviridae* family members [335]. This opens avenues for the evaluation of the anti-YFV activity of molecules that have been clinically approved for treatment against other *Flaviviridae* and notably, the hepatitis C virus (HCV). For instance, Sofosbuvir, a uridine nucleotide prodrug which targets the viral RNA polymerase and is clinically approved for use against HCV, has been shown to inhibit ZIKV replication efficiently in vivo, and prevented death in ZIKV-infected mice [336,337]. In addition, Sofosbuvir showed antiviral activity against DENV in vitro [338], so its potential as an anti-YFV compound is currently being investigated.

Host proteins that take part in the viral replication cycle constitute another pool of potential drug targets for anti-YFV molecule development. The purine nucleoside Ribavirin inhibits (among other potential modes of action) the inosine monophosphate dehydrogenase (IMPDH) protein and induces a reduction in the GTP pool, a mechanism that has been shown to correlate with its antiviral activity against several RNA viruses [339]. Ribavirin has shown anti-YFV activity in vitro and in vivo in hamsters, but not in NHPs [1,340,341]. The inhibition of the host caseine kinase 1, an NS5-interactant [342], and of both NTRK1 and MAPKAPK5 kinases, necessary for virus assembly, efficiently precluded YFV replication in vitro but none of these strategies has been tested in vivo yet [343].

Proteins involved in the host immunological response are also relevant targets for YF disease management as its most critical part, the shock (or intoxication) phase, has been suggested to have an immunopathological basis and is thought to involve a “cytokine storm” (i.e., unbalanced cytokine response). Several therapies have thus been developed to modulate this inflammatory response. Treatment with interferon-α significantly improved survival and reduced serum alanine aminotransferase levels in hamsters and African green monkeys when administered within 24 h following infection [1,340], showing a good potential for post-exposure prophylactic administration. The use of a recombinant adenovirus expressing interferon-α effected protection of hamsters against challenge with the hamster-virulent YF Jimenez strain up to two days post-infection [344]. Moreover, data from a retrospective analysis in patients having YF VAAE accompanied by shock, showed that treatment with stress-dose corticosteroid improved survival in humans [272,345]. These experimental and clinical insights strongly suggest that strategies counteracting cytokine storm and shock are efficient therapies for YF disease and should be further investigated.

## 4. Discussion

Control over YFV infections is a multifaceted issue to which no miraculous solution can be proposed for the time being. Indeed, curbing the circulation of a virus which circulates in the forest, in multiple non-human primate species, and which can be transmitted to humans through the bite of numerous mosquito species is a tremendous challenge. The fight against YFV—and numerous other zoonotic viruses—is like an arm-wrestling match during which every drop in effort, no matter how slight, constitutes, for the adversary, a chance to get the upper hand. This phenomenon can be appreciated in the pattern of shrinkage and expansion of YFV areas of endemicity according to vaccination and vector control campaigns across the last century of the fight against YFV [145,272].

Yellow fever activity has importantly increased in both Africa and South America over the past decades, as the result of insufficient vaccination coverage, mosquito reinfestation, deforestation and urbanisation. The fear of large urban YFV outbreaks has resurfaced in South America and with equal concern, numerous territories in the world that, until now, have apparently remained free of the virus, appear to bring together host(s), vector(s) and climatic conditions that are suitable for YFV dissemination [346]. In response to this resurgence, and to prevent the risk of expansion of YFV outbreaks to urban areas with dense non-immune populations, it is necessary to strengthen and refine surveillance, increase prevention through vaccination and vector-control and finally, improve patient care. Notably, from this perspective in 2017, the WHO initiated the “EYE” (Eliminate Yellow fever Epidemics) initiative in order to “protect the populations most at risk, ensure a ready supply of YF vaccine, build resilience in urban centres and prevent international spread” [347].

If we refer to the most recent outbreaks in Brazil and in Africa (e.g., in Nigeria, Angola, DRC, Chad, Sudan, Ethiopia), a likely explanation for the large number of cases that were reported is that the virus circulated in regions with low vaccination coverage [145]. Hence, we need to rethink how areas with vaccination recommendation are defined. In this prospect, it is necessary to improve our understanding of how the virus spreads and evolves.

Epidemiological surveillance is the main approach for tracing virus circulation and is primarily dependent on the availability of sensitive and specific diagnostic tools. As discussed in Section 3.1, there is still room for improvement in terms of sensitivity and specificity regarding diagnostic tools for YFV detection. In the case of YFV, these requirements come in a particularly intricate context as flaviviruses close to YFV in terms of serological and clinical features circulate in YFV endemic areas (e.g., Zika, Dengue) and as wild-type and vaccinal strains elaborated from YFV 17D (e.g., YFV 17DD or Dengvaxia^®^) should be accurately distinguished. Finally, in an outbreak context, diagnostic tools should be operable under field conditions.

Given the limited amount of genetic data available for YFV, it is necessary to constitute comprehensive catalogues of genomes of wild-type YF strains, notably during outbreaks, to better appraise YFV genetic diversity and develop comprehensive molecular detection systems. During the last Brazilian outbreak, efforts have been made to achieve genome sequencing using conventional PCR methods [133,139,348] and in the field, using portable sequencing tools, resulting in more than 50 additional genomic sequences now being available on Genbank for YFV [197]. To take full advantage of viral genetic data, they should be constantly confronted with the clinical and phenotypical features of the viruses in order to identify potential causal relationships and whenever possible, investigate them. It has been proposed that divergence between YFV genotypes would contribute to differential adaptation to mosquito vectors [113]. In the same vein, specific South American lineages have been suggested to be associated with increased epidemic potential (e.g., South American I modern lineage) [132,137]. However, no causal link has been demonstrated in either case.

Finally, it has been postulated that differences in transmission dynamics (e.g., sylvatic versus sylvatic/savannah cycle) or specific interactions with mosquito vectors (e.g., maintenance through a TOT mechanism) may result in different evolutionary dynamics among YFV genotypes [113]. Indeed, differences in maintenance mechanisms may modulate the selective pressures and the replication rate that dictate the evolution of the viral genome [108]. As its natural upkeep importantly relies on a sylvatic transmission cycle, YFV is considered to be a “sylvatic” virus. By contrast, DENV, the maintenance of which largely relies on an urban transmission cycle [349], is considered to be an “urban” virus, with a greater epidemic potential than YFV [350]. As discussed in Section 2.1.3, in terms of evolutionary dynamics, YFV exhibits a slower evolutionary trend when compared to DENV. As reported by Sall and colleagues [108], the evolutionary rate of DENV2 sylvatic genotype is the one which is closest to that of YFV and thus the lowest when compared to other DENV2 genotypes, with ~6 × 10^−4^ substitutions/site/year. Although this could be pure chance and if not, would possibly involve multiple factors, it could be interpreted as additional evidence that “sylvatic” behaviour may result in a lower evolutionary rate than an “urban” one. Overall, we need additional genetic and phenotypic data to properly determine whether there is a significant difference in evolutionary rate between sylvatic and non-sylvatic isolates of DENV and if an analogy cannot reliably be drawn between sylvatic DENV and YFV [351,352]. Further investigating the impact of specific maintenance patterns on the evolutionary behaviour of YFV may allow us to gauge the changing landscape within its reach and thereby, the epidemic potential of this virus.

In recent decades, increasing efforts have been implemented to expand vaccination coverage in at-risk areas and outbreak occurrence in densely populated areas have additionally increased the demand for YF vaccine. Hence, reconciling vaccine supply and demand is more than ever a central issue for YF prevention. For instance, the EYE initiative has planned to use 1.4 billion doses throughout the next eight years. This target would require the production of 175 million vaccine doses each year [353] but currently, the maximum yearly production of vaccine has never exceeded 80 million doses [353]. This challenge comes in a particularly difficult context of expansion of YFV-endemic areas and in which the connection between these regions and territories that are free of YFV—but suitable for virus transmission—is strengthening.

As evoked in this manuscript, several strategies have been proposed to increase the number of available vaccine doses by using reduced amount of vaccine through dose-fractioning and intradermal administration. To overcome the issue of supply it is necessary to increase gross vaccine production. The easiest option would be to have more doses produced each year, as planned by the EYE strategy, but this would not solve the problem of timeliness in replenishment in a context of successive large outbreaks, as observed during the past three years. To refine the vaccine manufacturing process, procedures could be revisited, as virus production technologies have evolved tremendously during the last decades. DENV (Dengvaxia) and JEV (Imojev) vaccines are cultured *in cellulo* rather than in embryonated chicken eggs, which is a much more efficient procedure [29]. Given that these vaccines have a YFV 17D backbone, it is likely that these protocols could successfully be adapted to overcome the difficulties previously encountered in the production of YFV 17D in cell culture [29,145]. Although revisiting manufacturing protocols is a laborious process, it will result in a more convenient, faster and more reliable production pipeline that will certainly benefit the overall disease prevention process. Furthermore, expertise from such developments should prove most useful for the implementation of more efficient and convenient vaccine manufacturing procedures in general.

Widening the use of YFV vaccine would also imply to improve management of VAAE. Although their occurrence can be considered as rare (~0.4 cases/100,000 cases), if 1.4 billion doses of the vaccine are to be used in the next eight years this would consequently lead to ~5600 adverse events that will have to be taken care of. In addition, if the EYE reaches its ambitious goal by 2026, until then, it is possible that other YFV outbreaks will occur. Therefore, it is still crucial to work on new antiviral strategies against YFV. Additional investigations should be made to identify the host factors that are necessary to the different steps of the YFV replication cycle (e.g., entry, replication, assembly, and egress) and to identify precisely, the host and viral factors that are involved in pathogenesis (e.g., immune response, entry pathway).

General questions deserve to be raised as the YFV vaccination campaigns that are to be implemented—notably through the EYE initiative—will be unprecedented in scale and may have several side effects that it would be worth anticipating as far as possible (e.g., exposure of immunocompromised individuals, impact on the circulation of other antigenically closely related flaviviruses, etc.). There is also an urgent need to improve our understanding of the vaccine itself (e.g., attenuation mechanism, efficient priming of a long-term immune response), the latter considerations have been concisely discussed in the recent work of Douam and Ploss [145].

Finally, increasing our knowledge of the molecular biology of wild-type YFV should benefit greatly to the research on other zoonotic viruses and more specifically, arboviruses. Furthermore, working on tools and strategies to contain YF outbreaks will be most useful to the development of countermeasures against other arboviral diseases notably flaviviruses, including pathogens of importance for Public Health at the moment such as DENV or ZIKV.
